# Supplementary material for: Interfacial Coupling and Electronic Structure of Two-Dimensional Silicon Grown on the Ag(111) Surface at High Temperature
Source: Sci Rep. 2015 Jun 18;5:10310. doi: 10.1038/srep10310 (PMC5155519; doi:10.1038/srep10310)
Supplement: Supplementary Information [file srep10310-s1.pdf]

Supplemental Information: Interfacial Coupling and Electronic Structure of Two-Dimensional Silicon Grown on the Ag(111) Surface at High Temperature

Jiagui Feng,[1] Sean R. Wagner,[1] and Pengpeng Zhang[1, a)]

[1] *Department of Physics and Astronomy, Michigan State University, East Lansing, Michigan 48824-2320, USA*

[a)]Electronic mail: zhang@pa.msu.edu

TOPOGRAPHY IMAGES AND HEIGHT MEASUREMENTS OF $(\sqrt{3} \times \sqrt{3})$ MULTILAYERS ON AG(111)

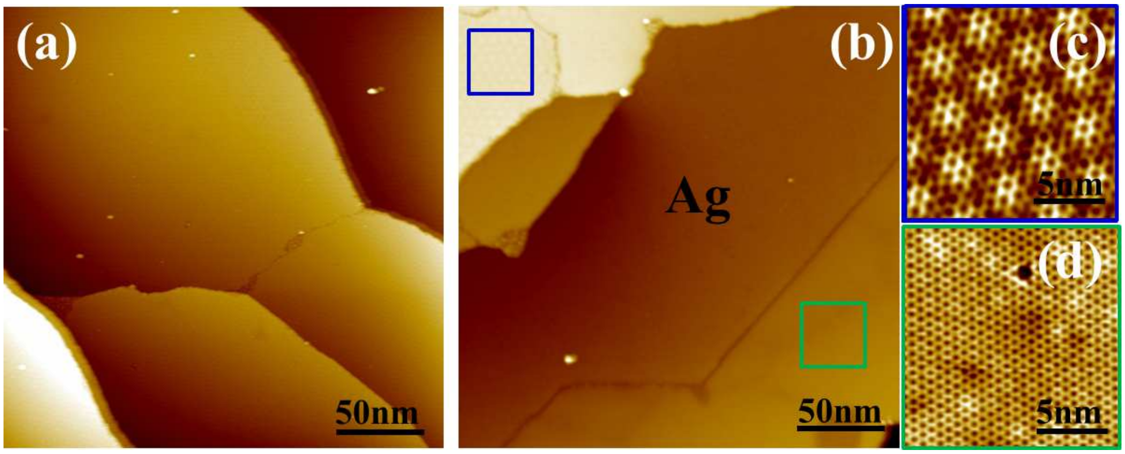

Figure S1 | STM topography images (V_s = -1..0 V; I_t = 50 pA) obtained at 77K on (a) a monolayer of Si $(\sqrt{7} \times \sqrt{7})$ superstructure covering nearly the entire Ag(111) surface and (b) the $(\sqrt{3} \times \sqrt{3})$ atomic layer along with the Si $(\sqrt{7} \times \sqrt{7})$ superstructure. (c) and (d) display zoomed in STM topography images (V_s = -1.0 V; I_t = 50 pA) obtained at 77K of the Si $(\sqrt{7} \times \sqrt{7})$ superstructure and the $(\sqrt{3} \times \sqrt{3})$ atomic layer on Ag(111). The colored outlines correspond to the colored boxed regions in (b).

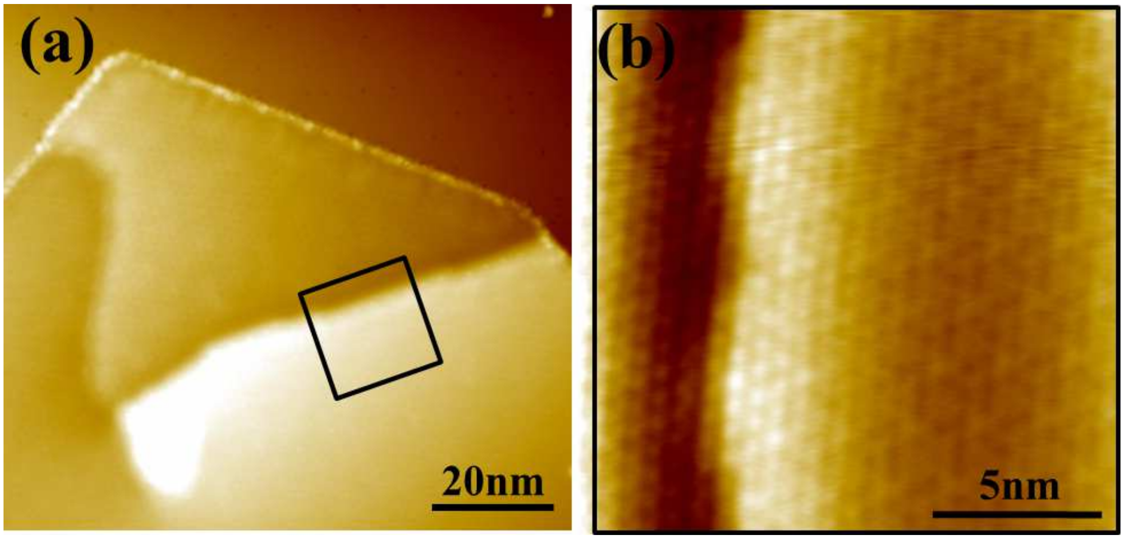

Figure S2 | (a) The same STM image as shown in Figure 1 (a) (V_s = +1.5 V; I_t = 50 pA) obtained at 77K. The film is grown on a defected area on the Ag(111) substrate. (b) Zoomed- in STM topography image (V_s = +0.5 V; I_t = 200 pA), obtained at 77K, of the boxed area in (a). It illustrates a continuous film conformal to the defect feature on the substrate.

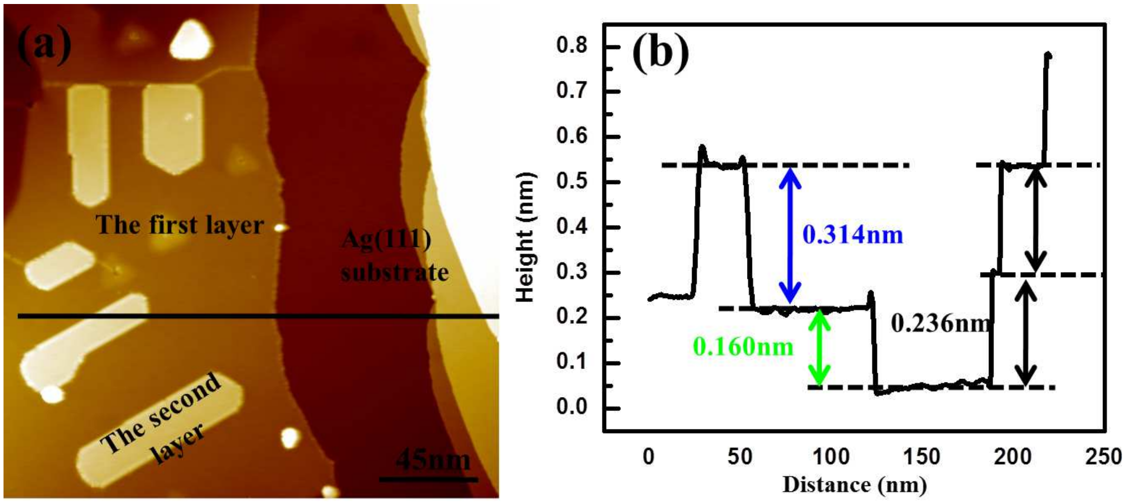

Figure S3 | STM topography image and line profile of multilayered ($\sqrt{3} \times \sqrt{3}$) phase on Ag(111) substrate obtained at 77K. (a) STM image showing the ($\sqrt{3} \times \sqrt{3}$) film grown on a clean Ag terrace without defects (V_s = +1.5 V; I_t = 50 pA). (b) Apparent height line profile taken along the black mark denoted in (a). The line profile shows the apparent interlayer spacing of Si structures (the blue arrow), the apparent height difference between the first ($\sqrt{3} \times \sqrt{3}$) atomic layer and the underlying Ag surface (the green arrow), and the apparent out-of-plane spacing of the Ag(111) substrate (the black arrows).

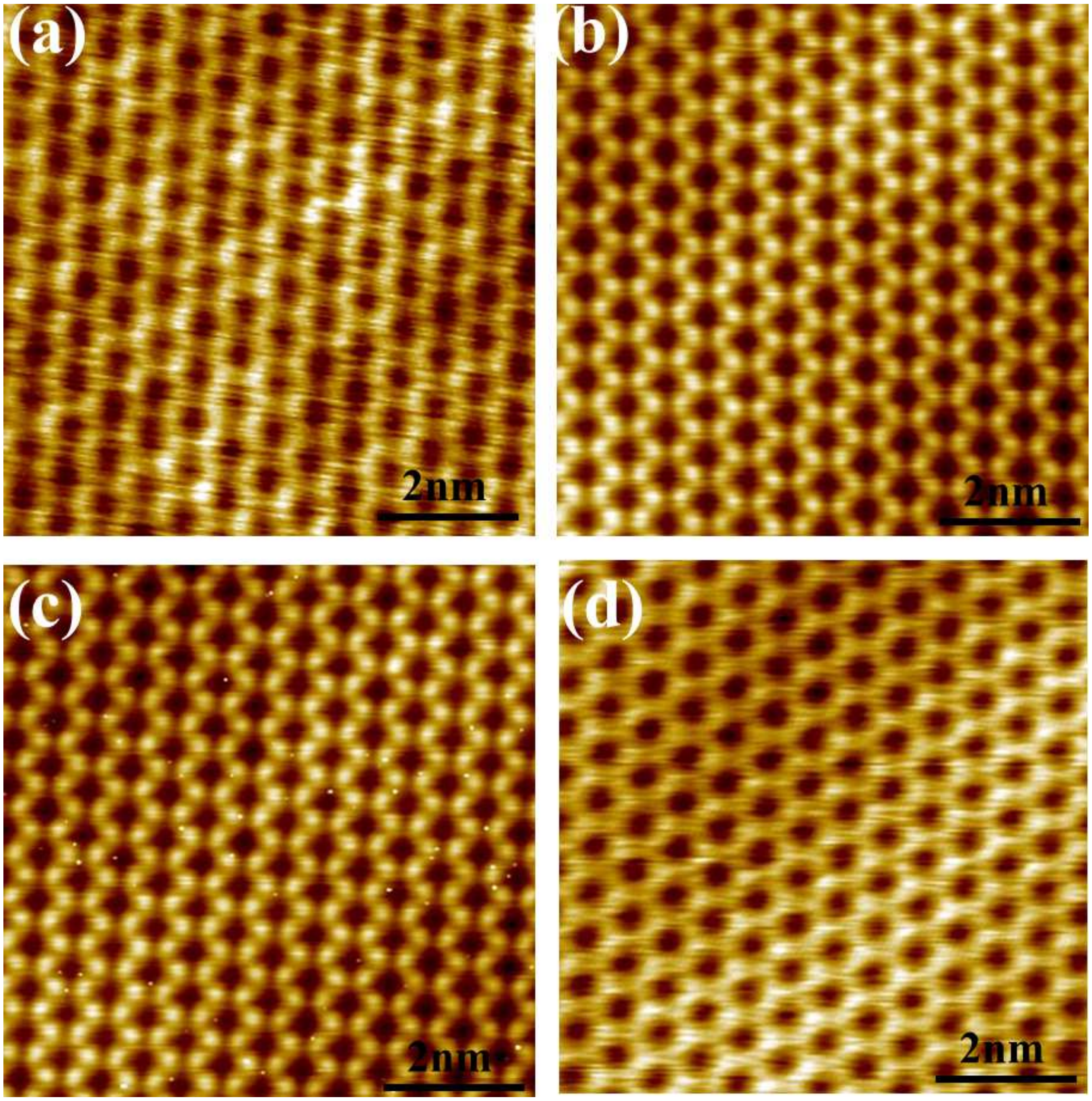

Figure S4 | (a)-(d) A series of STM topography images ($I_t = 50$ pA) obtained at 77K of the sixth atomic layer of the ($\sqrt{3} \times \sqrt{3}$) phase. Each image was obtained at different sample bias: (a) $V_s = +1.5$ V, (b) $V_s = +0.5$ V, (c) $V_s = +0.3$ V, and (d) $V_s = -1.0$ V. In these images of the thick multilayered film, only the top ($\sqrt{3} \times \sqrt{3}$) surface structure can be identified without any interference effects from the underlying interface structure.

GEOMETRIC STRUCTURES OF THE $(\sqrt{3}\times\sqrt{3})$ PHASE GROWN ON THE $(\sqrt{7}\times\sqrt{7})$ INTERFACIAL LAYER

Note that the $(\sqrt{7}\times\sqrt{7})$ superstructure observed in our experiments is indexed with regard to the Si lattice. It is equivalent to the $(2\sqrt{3}\times 2\sqrt{3})R30^{\circ}$ structure with respect to the Ag lattice.

To be more precise, this structure should be expressed as $(\sqrt{7}\times\sqrt{7})$R$\pm$19.1°. The angle of the structure is referenced relatively to the Si[110] directions that are rotated from the Ag[110] direction by $\pm 10.9^{\circ}$, as illustrated in the schematics in Fig. S6(e). Thus, the geometric structures of the $(\sqrt{3}\times\sqrt{3})R30^{\circ}$ domains subsequently grown on top of the $(\sqrt{7}\times\sqrt{7})$ superstructure can be configured by the rotation angles between the two lattices, i.e., *i*) +30°/-19.1° (+49.1°); *ii*) -30°/-19.1° (-10.9°); *iii*) -30°/+19.1° (-49.1°); and *iv*) +30°/+19.1° (+10.9°). Due to the symmetry of the lattices, *i*) and *ii*) are essentially equivalent, as well as *iii*) and *iv*), leading to only one allowable $(\sqrt{3}\times\sqrt{3})$ domain on each of the mirror-reflected $(\sqrt{7}\times\sqrt{7})$R$\pm$19.1° superstructures. These two $(\sqrt{3}\times\sqrt{3})$ domains are shown in Fig. S6(a) and (c). And from the corresponding fast Fourier transforms (FFTs) (Fig. S6(b) and (d)) we determine that the two domains are rotated from each other by 21.8°, consistent with the geometry illustrated in Fig. 6(e). Lastly, the simulated geometric structure and the surface diffraction pattern from these assignments have successfully predicted the configuration of the Moiré patterns which match closely with the experimental observation. The FFTs shown in Fig. S6(b) and (d) also illustrate that the $(\sqrt{21}\times\sqrt{21})$ Moiré pattern remains the same orientation on the two 3 domains, as expected from the results presented in Fig. S5 and Fig. S6(e).

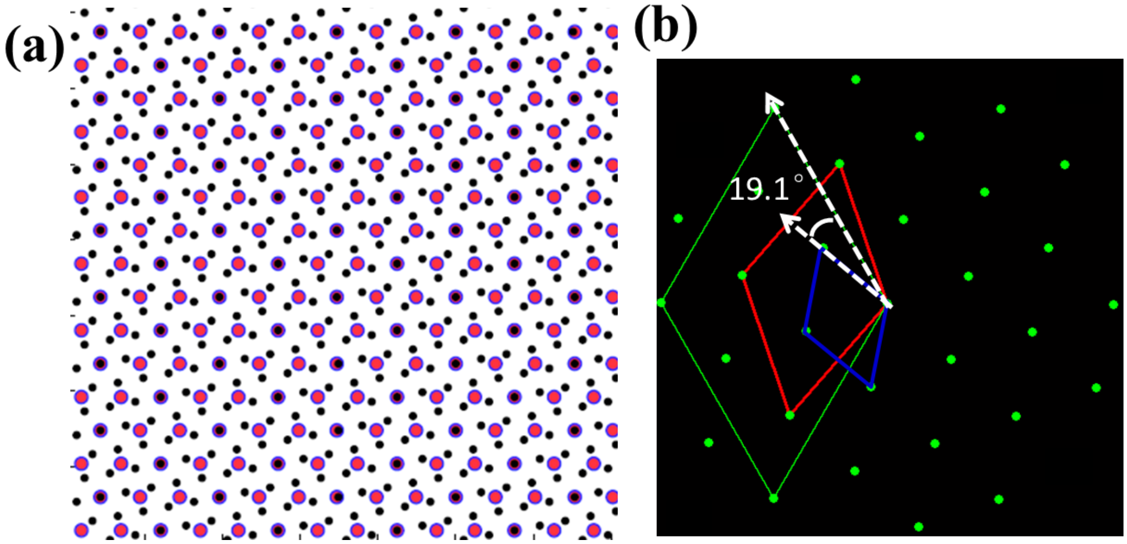

Figure S5 | (a) Simulated geometric structure of the lattice matching of the $(\sqrt{3} \times \sqrt{3})$ surface (black circles) rotated by 10.9° with respect to the underlying Si/Ag interface. The interface consists of a monolayer of Si in the honeycomb lattice (Si(1×1)) rotated with respect to the Ag(111) substrate surface, resulting in select Si atoms resting on top of underlying Ag atoms giving rise to the $(\sqrt{7} \times \sqrt{7})$ superstructure (indexed to the Si lattice). This superstructure is represented by the red circles outlined in blue. When the black circles of the $(\sqrt{3} \times \sqrt{3})$ structure are directly on top of the $(\sqrt{7} \times \sqrt{7})$ superstructure, the $(\sqrt{21} \times \sqrt{21})$ Moiré pattern is formed. (b) Simulated surface diffraction pattern of the $(\sqrt{3} \times \sqrt{3})$ structure rotated by 10.9° with respect to the underlying Si/Ag interface. Multiple scattering between the Ag(111), Si(1×1), and $(\sqrt{3} \times \sqrt{3})$ lattices are allowed. The $(\sqrt{3} \times \sqrt{3})$ structure (green), $(\sqrt{7} \times \sqrt{7})$ superstructure (red), and $(\sqrt{21} \times \sqrt{21})$ Moiré pattern (blue) display diffraction peaks in the pattern which allow for the appropriate unit cell assignment and comparison with the FFT insets in Fig. 2. The simulated surface diffraction pattern displays the same rotation angle of 19.1° between the $(\sqrt{21} \times \sqrt{21})$ Moiré pattern and the $(\sqrt{3} \times \sqrt{3})$ structure as observed in STM.

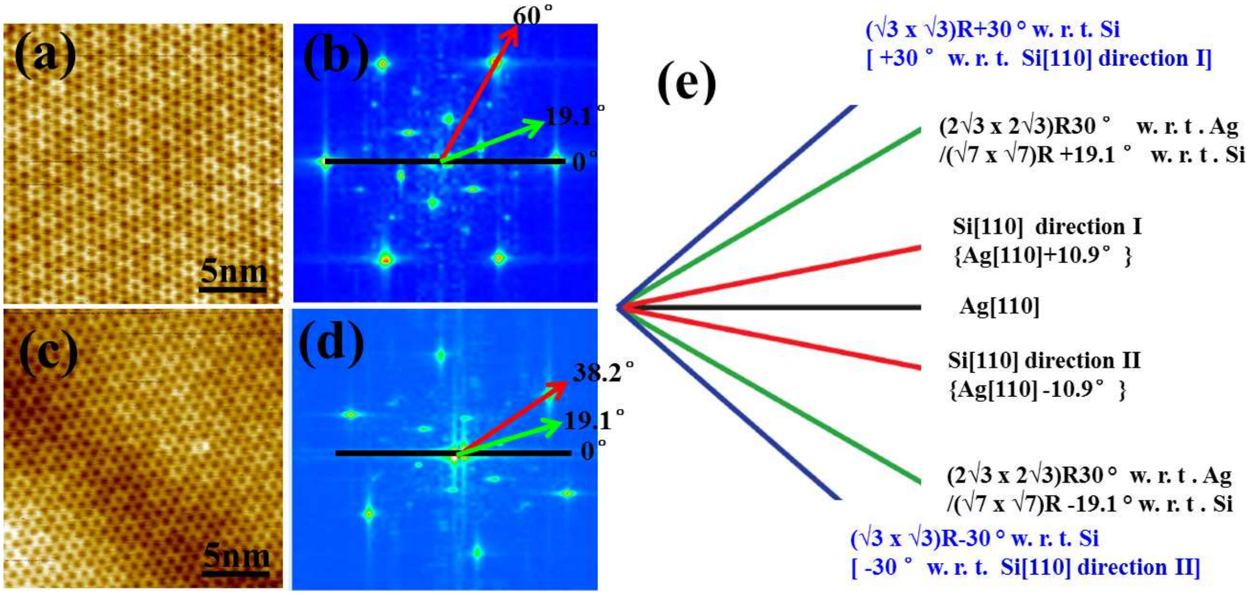

Figure S6 | STM topography images and FFTs of the two $(\sqrt{3} \times \sqrt{3})$ domains grown on the $(\sqrt{7} \times \sqrt{7})$ superstructure on Ag(111), obtained at 77K. (a) and (c) STM topography images of the two allowable $(\sqrt{3} \times \sqrt{3})$ domains on the $(\sqrt{7} \times \sqrt{7})$ superstructure on Ag(111) ($V_s = +1.5$ V; $I_t = 50$ pA). (b) and (d) are the corresponding FFTs of (a) and (c), respectively. In the FFT images, the red arrows represent the orientations of the $(\sqrt{3} \times \sqrt{3})$ domains with respect to the scanning direction (the black line). The two domains are rotated from each other by 21.8°. The green arrows show the orientations of the $(\sqrt{21} \times \sqrt{21})$ Moiré patterns, which remain the same on the two $(\sqrt{3} \times \sqrt{3})$ domains. (e) Schematics illustrating the angles of the $(\sqrt{3} \times \sqrt{3})$ R30° and the $(\sqrt{7} \times \sqrt{7})$R±19.1° structures relative to the Ag[110] and Si[110] directions, adapted from the reference arXiv:1412.4902.

STANDING WAVE OSCILLATIONS ON A VARIETY OF $(\sqrt{3} \times \sqrt{3})$ STRUCTURES

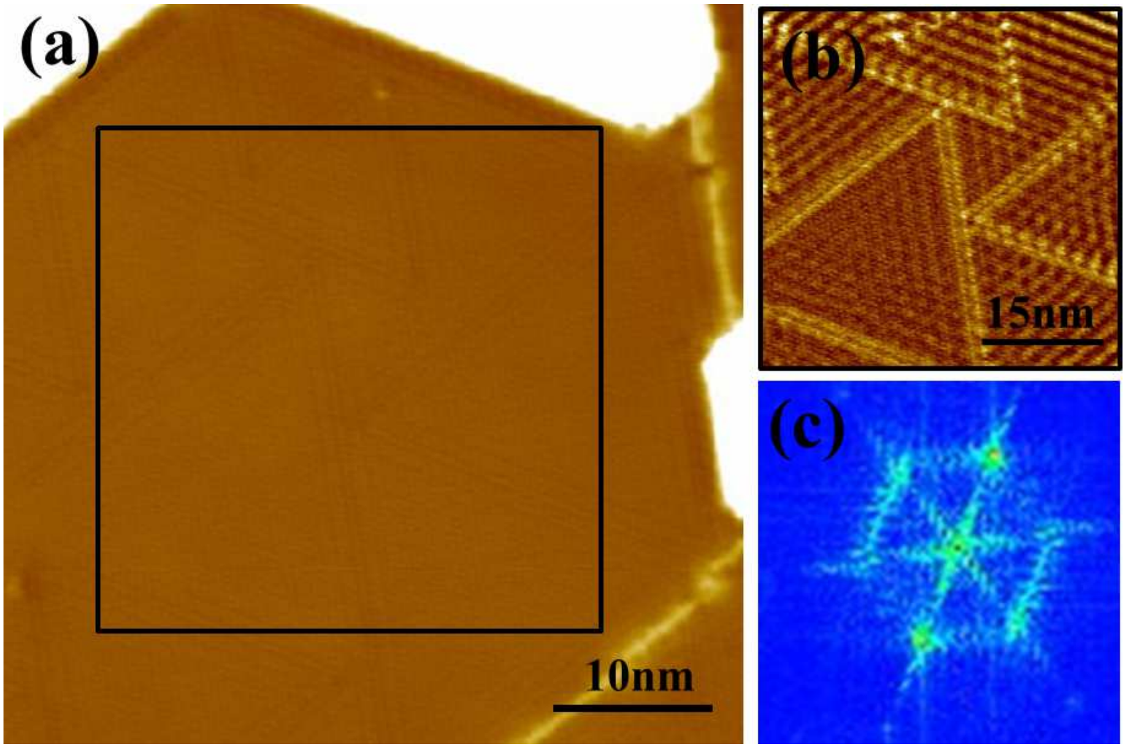

Figure S7 | Layout of the step edges participating in the electron scattering process and the resulting surface standing wave pattern. (a) Zoomed-out STM topography image (V_s = +1.5 V; I_t = 80 pA) of the area presented in Figure 4(a)-(f), obtained at 4.5K. The step edges that participate in the electron scattering process are clearly depicted. (b) Differential conductance (dI/dV) mapping (V_s = +0.8 V; I_t = 300) illustrating the electron standing wave pattern resulted from the electron scattering at the step edges shown in (a). (c) FFT image corresponding to the dI/dV mapping as shown in (b). The wave number is determined from the radial distance of the bright spots.

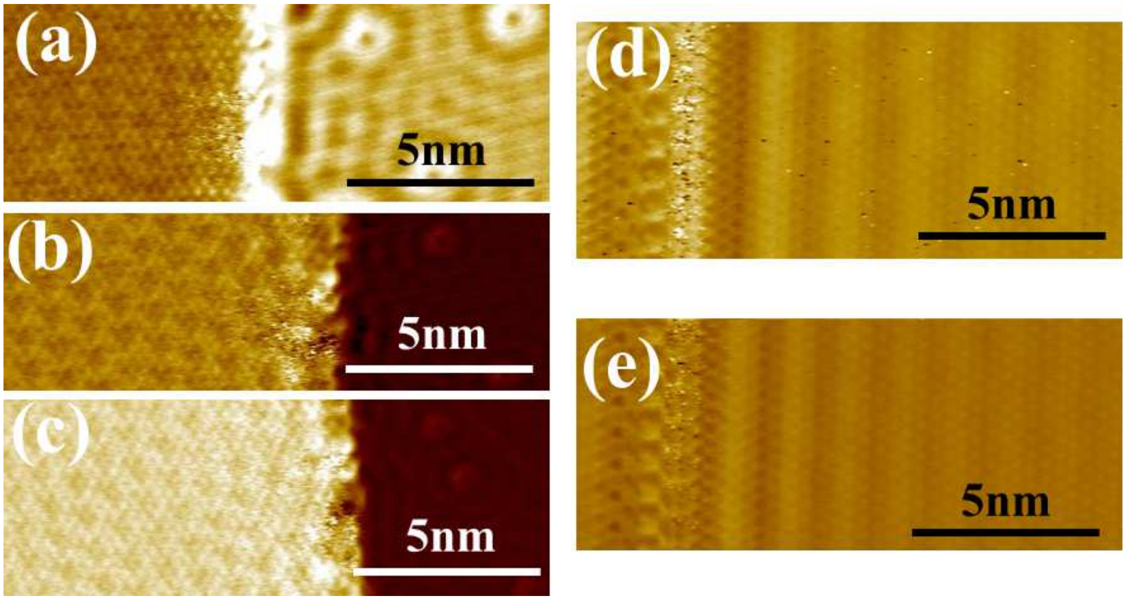

Figure S8 | (a)-(c) Differential conductance mapping images (I_t = 300 pA) obtained on the first ($\sqrt{3} \times \sqrt{3}$) atomic layer and the exposed Ag(111) substrate at 77K. Each image was obtained at different sample bias: (a) V_s = +0.3 V, (b) V_s = +0.7 V, and (c) V_s = +0.9 V. Unlike the case for Ag(111) the standing wave oscillation on the first ($\sqrt{3} \times \sqrt{3}$) atomic layer is too weak to be readily distinguished. (d) and (e) are differential conductance mapping images (I_t = 300 pA) obtained at 77K of the sixth ($\sqrt{3} \times \sqrt{3}$) atomic layer where the sample bias is V_s = +0.3 V in (d) and V_s = +0.5 V in (e). The multilayered films display standing wave oscillations with a large magnitude and a long decay distance.

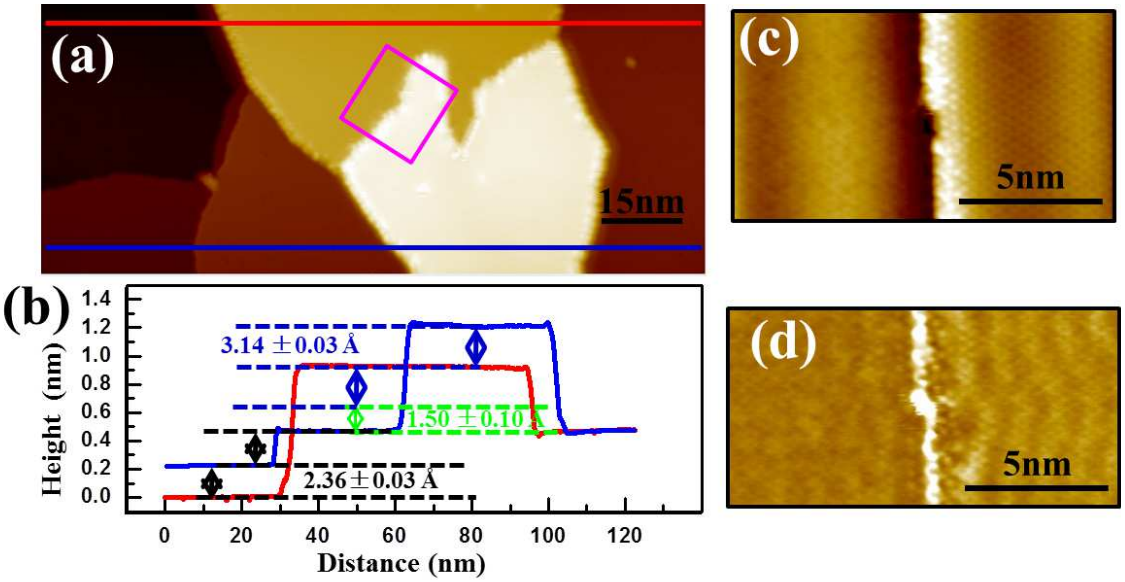

Figure S9 | (a) STM topography image of the Si/Ag surface (V_s = +1.5 V; I_t = 50 pA) which contains a multilayered ($\sqrt{3} \times \sqrt{3}$) island obtained at 77K. The boxed region is used as reference locations for zoomed-in STM topography images in Fig. 5. The red and blue lines in (a) denote the locations of the apparent height line profiles shown in (b) with the appropriate corresponding color. The line profiles show the apparent interlayer spacing of Si layers (blue arrows), the apparent height difference between the first ($\sqrt{3} \times \sqrt{3}$) atomic layer and the underlying Ag surface (the green arrow), and the apparent out-of-plane spacing of the Ag(111) substrate (black arrows). The interlayer spacing of Si films matches with the bulk Si(111) d-spacing. (c) and (d) are simultaneously obtained STM topography and differential conductance images (V_s = +0.5 V; I_t = 300 pA) taken on the second and third ($\sqrt{3} \times \sqrt{3}$) atomic layers. The upper layers display enhanced standing wave oscillations.

EVALUATION ON THE STRENGTH OF THE SCATTERING BARRIER AT THE VACUUM-FILM VS THE FILM-FILM EDGE

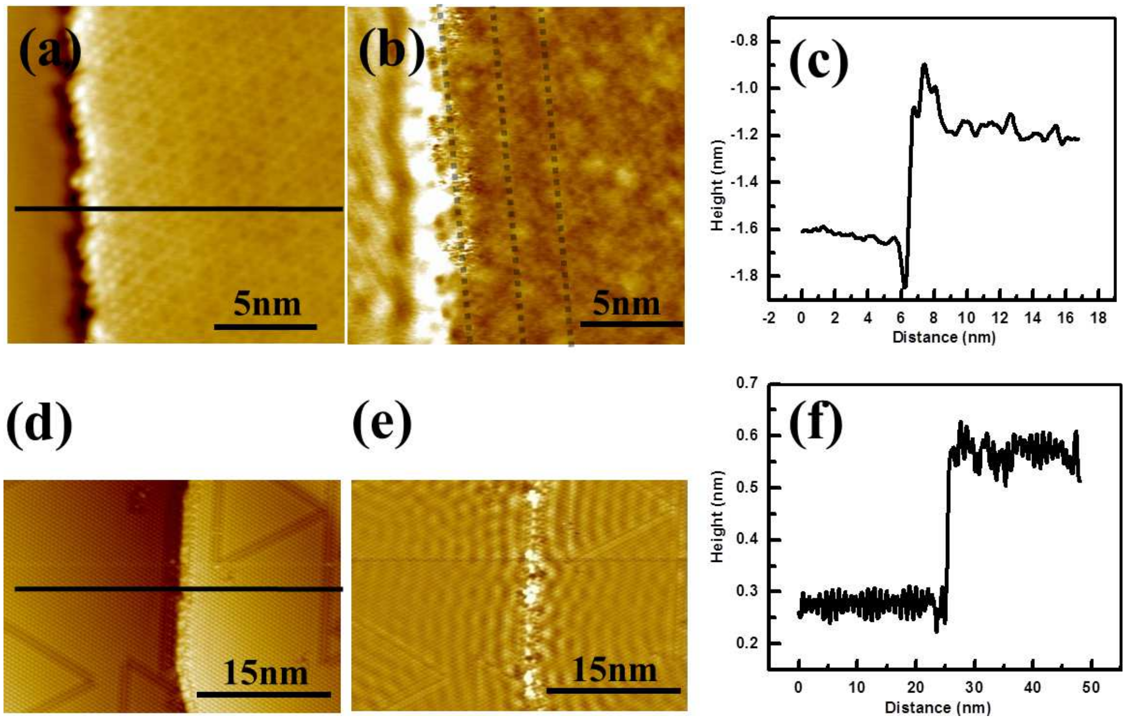

Figure S10 | (a) and (b) are simultaneously obtained STM topography and differential conductance mapping images ($V_s = +0.5$ V; $I_t = 300$ pA) taken on the second $(\sqrt{3} \times \sqrt{3})$ atomic layers, obtained at 77K. The three weak standing wave oscillation peaks on the film surface are marked by the dotted lines to guide the view. (c) Apparent height line profile taken along the black line denoted in (a). The apparent height difference between the film and the underlying Ag surface is 0.473±0.003nm. (d) and (e) are simultaneously obtained STM topography and differential conductance mapping images ($V_s = +0.5$ V; $I_t = 300$ pA) taken on a multilayered Si film ($>$ 6 atomic layers) with $(\sqrt{3} \times \sqrt{3})$ reconstructed surface, obtained at 4.5K. The magnitude and decay length of the standing wave oscillations on the upper and lower terraces are comparable to each other. (f) Line profile taken along the black mark denoted in (d). The apparent interlayer spacing of Si structures is 0.314±0.003nm, consistent with the d-spacing of bulk Si(111).

To evaluate the strength of the scattering barrier at the vacuum-film vs. the film-film edge, we compare the magnitude and decay length of the standing wave oscillations on the N and N+1 layers of a thick film where the layer-dependent variation due to the substrate

influence is completely suppressed. On the Nth layer, the standing wave will encounter scattering at the film-film edge, whereas on layer N+1 the scattering barrier at the vacuum-film edge becomes relevant. If these two scatting barriers are of comparable strength, we expect comparable standing wave decay length on the N and N+1 layers. And indeed this is what we observe in Fig. S10(d) and (e). Alternatively, we can compare the standing wave decay on a specific $(\sqrt{3} \times \sqrt{3})$ atomic layer, for instance, the second layer, imposed by either the vacuum-film edge (Fig. S10(b)) or by the film-film edge (the lower terrace in Fig. 5(d)). In both cases, we can identify three oscillation peaks in the dI/dV mapping, suggesting comparable decay lengths although the standing waves have been scattered by two different barriers.
